# Occurrence of idiopathic pulmonary fibrosis during immunosuppressive treatment: a case report

**DOI:** 10.1186/s13256-016-0916-5

**Published:** 2016-05-25

**Authors:** Stefania Cerri, Giacomo Sgalla, Luca Richeldi, Fabrizio Luppi

**Affiliations:** 1 Center for Rare Lung Diseases, University Hospital Policlinico di Modena, Via del Pozzo, 71-41124 Modena, Italy; 2 National Institute for Health Research Respiratory Biomedical Research Unit, Mailpoint 813, LE75 E Level, South Academic Block, Southampton, UK; 3 University Hospital Southampton NHS Foundation Trust, Southampton, SO16 6YD UK

**Keywords:** Idiopathic pulmonary fibrosis, Immunosuppressant, Steroids, Heart transplantation, Treatment

## Abstract

**Background:**

Immunosuppressive therapy has been—until the recent release of new guidelines on diagnosis and management—the recommended treatment for idiopathic pulmonary fibrosis. However, its efficacy in patients with idiopathic pulmonary fibrosis has always been a matter of debate.

**Case presentation:**

We report the occurrence of idiopathic pulmonary fibrosis in a white man receiving chronic immunosuppressive treatment following a heart transplant.

**Conclusions:**

This case report suggests that the immune mechanisms targeted by azathioprine and cyclosporine do not play a role in the pathogenesis of idiopathic pulmonary fibrosis.

## Background

Idiopathic pulmonary fibrosis (IPF) is a devastating lung disorder with a median survival of 3.5 years from diagnosis [1]. Until the recent release of the American Thoracic Society/European Respiratory Society/Japanese Respiratory Society/Latin American Thoracic Association guidelines [2], the standard of care for IPF consisted of corticosteroids and azathioprine in combination, although clinical practice suggests that immunosuppression does not impact the survival of patients with IPF. In addition, the authors of an interim analysis of a large, multicenter, placebo-controlled study [3] warned that a treatment regimen including prednisone and azathioprine is harmful for patients with IPF. Indeed, in that study sponsored by the National Heart, Lung, and Blood Institute, patients with IPF receiving a triple therapy with prednisone, azathioprine, and *N*-acetylcysteine showed higher mortality (11 %) than those receiving placebo (1 %). In this case report, we discuss the occurrence of IPF in a patient receiving chronic immunosuppressive treatment following a heart transplant.

## Case presentation

Our patient was a 75-year-old white man who was a former smoker (20 pack-years) with no relevant family history of lung disease. He had been a farmer until the age of 40 years. At the age of 42 years, he had an acute myocardial infarction, which led to the development of chronic heart failure with recurrent episodes of pulmonary edema. His medications included acetylsalicylic acid (150 mg/day) and amiloride/hydrochlorothiazide (5/50 mg/day, respectively). He had never received antiarrhythmic drugs, including amiodarone. Due to worsening clinical conditions, at the age of 54 years he was included on a heart transplant list and after 2 years on the list he underwent a heart transplant. At that time, his chest x-ray and lung function test results, including forced vital capacity (FVC) and diffusing capacity of the lungs for carbon monoxide (DL_CO_), were normal.

The patient, who was already being treated with felodipine (5 mg/day) and atorvastatin (5 mg/day), was initially started on azathioprine (50 mg twice daily), cyclosporine (100 mg twice daily) and prednisone, with the first two drugs being maintained in the long-term treatment. At the age of 65 years, following a chest trauma, he underwent chest radiography, which revealed the presence of bilateral reticulonodular abnormalities predominant in the lower lobes. Chest high-resolution computed tomography confirmed the presence of bilateral reticular abnormalities with minimum honeycombing in the periphery of both lower lobes. Chest auscultation revealed bibasal pulmonary “velcro” crackles. Lung function tests showed a mild restrictive ventilatory defect: FVC 73 % of predicted, forced expiratory volume in 1 second 75 % of predicted, total lung capacity 67 % of predicted, and DL_CO_ 60 % of predicted. The patient was completely asymptomatic. Bronchoalveolar lavage (BAL) cytology showed a slight lymphocytosis with prevalence of CD4^+^ T lymphocytes and a CD4/CD8 ratio of 3.6. Direct stains and cultures for bacteria, fungi, and mycobacteria on BAL samples were negative. Serum and BAL precipitins were negative.

**Fig. 1:**
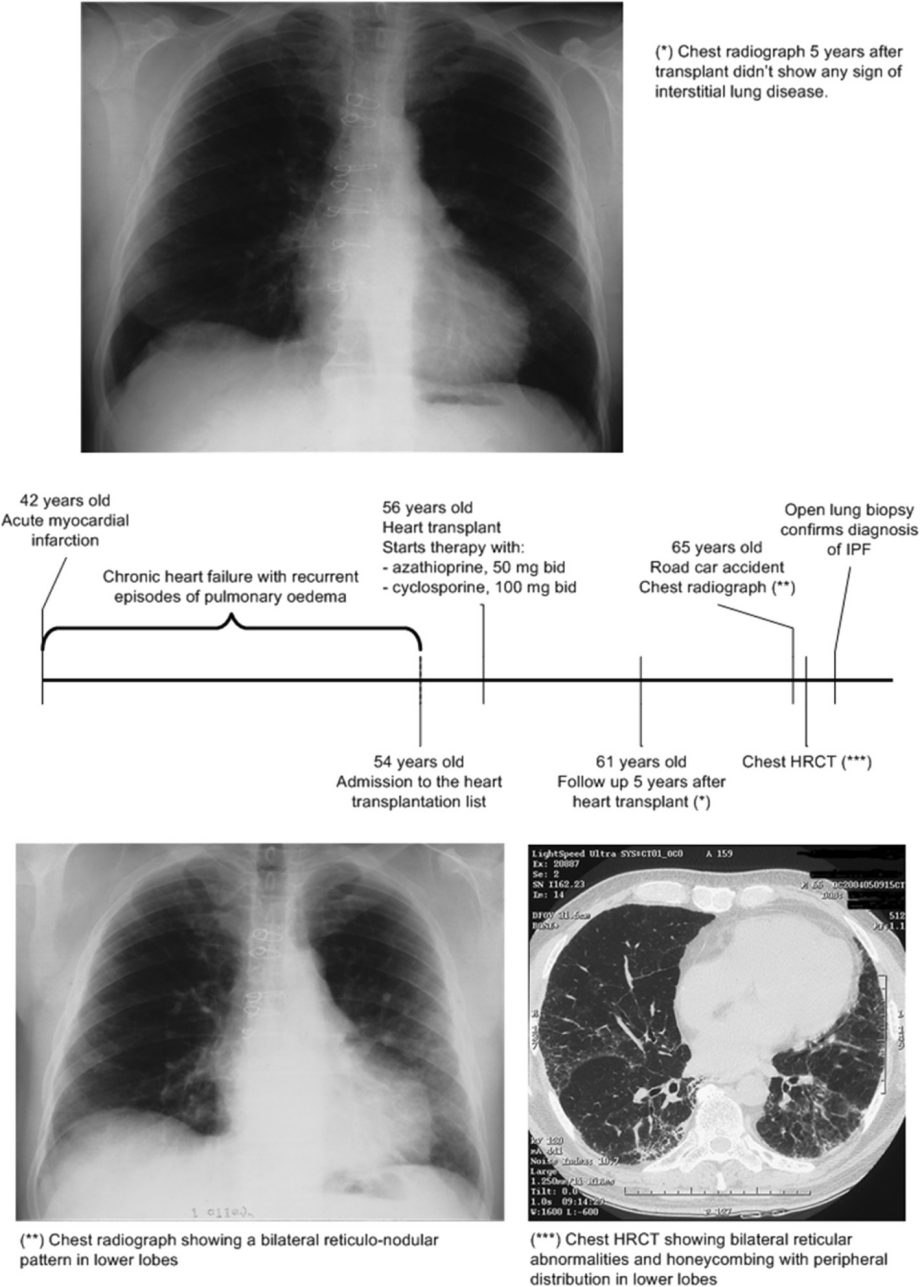
Timeline of the patient’s clinical history. *HRCT* high-resolution computed tomography, *IPF* idiopathic pulmonary fibrosis

To confirm the suspicion of IPF, an open lung biopsy with sampling in the right lower and middle lobes was performed. The histological pattern was consistent with usual interstitial pneumonia (UIP), thus providing, after exclusion of secondary causes and in the absence of elements for relevant professional and/or environmental exposures, the definite diagnosis of IPF was made 10 years after initiation of immunosuppressive therapy. While continuing immunosuppressive treatment, the patient’s clinical conditions progressively worsened (FVC 44 % predicted and DL_CO_ 4 % predicted), as expected in IPF. He died as a result of respiratory failure 5 years after receiving his diagnosis of IPF (Fig. 1).

## Discussion

Immunosuppression has long been the mainstream IPF treatment, despite scarce evidence of an impact on the natural history of the disease [4]. In fact, according to recent advances in IPF pathobiology, inflammation plays a minor—if any—role in the development of the disease [5, 6]. Azathioprine, an antimetabolite that exerts its immunosuppressive effects through the block of several T-cell functions, the inhibition of antibody synthesis, and the reduction of circulating monocytes and granulocytes [7], was, in combination with corticosteroids, the recommended treatment for IPF until the recent release of evidence-based guidelines on IPF. Nowadays, the mainstream IPF treatment is based on drugs with antifibrotic activity, such as pirfenidone and nintedanib, which have shown meaningful slowing of the rate of disease progression as measured by the rate of FVC decline [8, 9].

We report the occurrence of IPF in a patient on long-term azathioprine treatment in whom other known causes of pulmonary fibrosis with a histological pattern of UIP were excluded. In fact, although the patient worked as a farmer until the age of 40 years, a diagnosis of chronic hypersensitivity pneumonitis (HP) in our patient is unlikely. Particularly, histology showed a definite UIP pattern without any of the “ancillary findings” that are usually observed in patients with chronic HP, such as granulomas, eosinophils, and “bridging fibrosis.” Furthermore, his serum and BAL precipitins were negative.

As far as exclusion of possible drug-induced interstitial lung disease (ILD), we acknowledge that statin use may perhaps increase the risk of developing radiographic evidence of ILD, including findings characteristic of pulmonary fibrosis [10]; however, the occurrence of statin-induced ILD with a definite UIP pattern is not definitively established and is unlikely. Furthermore, there are no reports on atorvastatin in this clinical setting.

The occurrence of pulmonary interstitial pneumonitis during azathioprine treatment is well described [11–14]. However, azathioprine-induced pulmonary toxicity in the form of interstitial pneumonitis is commonly an acute or subacute event occurring early after treatment is initiated (ranging from 6 weeks to 1 year). Bedrossian and coworkers [11] reported the presence of a UIP-like pattern on lung biopsies of five patients taking azathioprine after kidney transplant, within 1 year after initiation of treatment. Three of the patients died due to acute respiratory distress syndrome a few days after lung biopsy, one patient died as a result of disseminated *Aspergillus* infection 30 days after biopsy, and one patient survived and recovered after azathioprine discontinuation and institution of cyclophosphamide therapy. Therefore, to the best of our knowledge, no sure cases of IPF fulfilling the currently accepted diagnostic criteria for the disease have been described during treatment with azathioprine.

## Conclusions

Although we cannot exclude the presence of early subclinical IPF at the time of heart transplant, our patient’s case provides indirect evidence that the mechanisms affected by conventional immunosuppressive therapies do not play a major role in the pathogenesis of IPF. Moreover, we cannot exclude that in this patient immunosuppressive treatment accelerated or worsened lung function deterioration in line with the data provided by the Prednisone, Azathioprine, and *N*-acetylcysteine in Patients with IPF study [3].
